# Unravelling the effect of the Dutch school-based nutrition programme Taste Lessons: the role of dose, appreciation and interpersonal communication

**DOI:** 10.1186/s12889-016-3430-1

**Published:** 2016-08-05

**Authors:** Marieke C. E. Battjes-Fries, Ellen J. I. van Dongen, Reint Jan Renes, Hante J. Meester, Pieter van’t Veer, Annemien Haveman-Nies

**Affiliations:** 1 Division of Human Nutrition, Wageningen University, P.O. Box 8129, 6700 EV Wageningen, The Netherlands; 2 Division of Strategic Communication, Wageningen University, 6700 EW Wageningen, The Netherlands; 3 Steunpunt Smaaklessen & EU Schoolfruit, Division of Food Chemistry, Wageningen University, 6700 AA Wageningen, The Netherlands

**Keywords:** Nutrition education, Primary school, Effect evaluation, Implementation, Process indicators

## Abstract

**Background:**

To unravel the effect of school-based nutrition education, insight into the implementation process is needed. In this study, process indicators of Taste Lessons (a nutrition education programme for Dutch elementary schools) and their association with changes in behavioural determinants relevant to healthy eating behaviour are studied.

**Methods:**

The study sample consisted of 392 Dutch primary school children from 12 schools. Data were collected using teacher and child questionnaires at baseline, and at one and six months after the intervention. Multilevel regression analyses were conducted to study the association between dose, appreciation and children’s engagement in interpersonal communication (talking about Taste Lessons with others after the lessons), and change in knowledge, awareness, skills, attitude, emotion, subjective norm and intention towards two target behaviours.

**Results:**

With an average implementation of a third of the programme activities, dose positively predicted change in children’s subjective norm of the teacher after one month. Teachers and children highly appreciated Taste Lessons. Whereas teacher appreciation was inversely associated, child appreciation was positively associated with children’s change in awareness, emotion and subjective norm of teachers after one month and in attitude and subjective norm of parents after six months. Interpersonal communication was positively associated with children’s change in five determinants after one month and in attitude and intention after six months.

**Conclusions:**

The implementation process is related to the programme outcomes of Taste Lessons. Process data provide valuable insights into factors that contribute to the effect of interventions in real-life settings.

**Electronic supplementary material:**

The online version of this article (doi:10.1186/s12889-016-3430-1) contains supplementary material, which is available to authorized users.

## Background

School settings seem to be an effective environment for teaching children about healthy nutrition [1–3]. Therefore a wide variety of school-based nutrition interventions have been developed in the last few decades, showing varying results [1–6]. Few studies on nutrition education programmes have studied the influence of the implementation process on the intervention outcomes [7–12]. In evaluation settings where programme delivery is not controlled by researchers and thus will vary between different intervention sites, it is especially important to relate process indicators to effect outcomes in order to obtain insight into which factors influenced the obtained results [2, 5, 12–16].

Delivered dose, acceptability (appreciation) and integrity (fidelity) are perceived as the most important process indicators [13, 15–17]. In addition, teacher-related factors, adaptations to the programme and the quality of the process (such as attention and engagement) are perceived to influence the effect of interventions [12, 15, 16]. Some studies have investigated the association between one or more of these process indicators and the outcome of school-based nutrition interventions, focusing mainly on fruit and vegetable intake [7–11]. The Pro Children study showed a positive association between delivered dose and change in fruit and vegetable intake [7]. In three other studies however, no such relation was found [8–10]. Children's appreciation of Pro Children and Fruits and Vegetables Make the Marks showed positive associations with change in fruit and vegetable intake [7, 8], whereas teacher appreciation and student appreciation of Project Tomato were not related to change in fruit and vegetable intake [11]. The observed fidelity of the Gimme 5 programme was not significantly associated with fruit and vegetable intake [9].

From research on the effects of mass media campaigns, it seems that the process indicator *interpersonal communication* can be an important factor for obtaining behavioural change, representing the extent to which people talk about the programme [18–20]. Children may tell one another whether they liked the programme and discuss what they learned [20]. This kind of communication may enhance the effect of the message on children’s attitudes, intentions and behaviour [19, 20], possibly by memory facilitation, persuasive influence, social support and increases in self-efficacy [19]. So far, no study on school-based nutrition programmes has included children’s engagement in interpersonal communication as a process indicator.

The current study focuses on the nutrition education programme Taste Lessons (*Smaaklessen*) for Dutch elementary schools, which aims to interest children in taste, health and food quality. Outcome evaluation of Taste Lessons showed significant positive effects on several behavioural determinants towards tasting unfamiliar foods and eating healthy and a variety of foods [21]. To investigate how the implementation process contributed to these programme outcomes, the current study aims to provide insight into the programme delivery by studying delivered dose, appreciation and interpersonal communication, and to assess the association between these three process indicators and seven behavioural determinants relevant to tasting unfamiliar foods and eating healthy and a variety of foods.

## Methods

### Intervention design

Taste Lessons is a national school-based nutrition education programme for grades 1–8 (4–12-year-olds) in elementary schools [22]. It has been developed in practice, without using a particular theoretical framework. As the programme aims to increase children’s interest in food and to increase their knowledge and skills regarding healthy and conscious eating behaviour, children exposed to Taste Lessons are expected to increase in psychosocial determinants of healthy and conscious eating behaviour. During an introductory workshop, teachers are trained in how to implement the lessons, and they receive a toolkit with teacher manuals and materials. The programme consists of 10–12 lessons per two grades, with lesson length ranging from 45 min to 2 h. Each lesson has 3–9 standard and 1–6 optional activities that teachers can select to implement in their classroom, including taste-testing, conducting experiments and homework assignments.

### Study population

The study population consisted of Dutch elementary schools that registered for Taste Lessons and attended an introductory workshop between September and November 2011. During the workshop, these schools were invited by a member of the research team to participate if they intended to teach Taste Lessons in grades 5–8 (8–12-year-olds), had not implemented Taste Lessons before and did not intend to participate in another nutrition-related programme. Twelve out of 37 schools were willing to participate and met the inclusion criteria (25 classes, 560 children). After the baseline measurement, four classes from different schools decided not to implement Taste Lessons in the relevant school year. The 392 remaining children from 21 classes completed the effect evaluation questionnaires at the first follow-up measurement (70 % of the children in the baseline group). As the second follow-up measurement took place in the next school year, grade 8 children started that year in the first grade of secondary school and were excluded from the second follow-up measurement for practical reasons. Therefore, 296 children from 18 classes completed the effect evaluation questionnaire at the second follow-up measurement (53 % of the children in the baseline group). The process evaluation questionnaires were completed at the first follow-up measurement by 18 out of 20 teachers (one teacher gave lessons to two classes) and the child appreciation forms by 339 of the 392 children (86 %).

### Study design and procedure

The current study was part of a larger effect evaluation with a quasi-experimental design and was carried out among twelve schools that implemented Taste Lessons in the 2011–2012 school year. Data were collected by means of questionnaires at baseline (September–December 2011), one month after the intervention and six months after the intervention (February–June and September–December 2012). For data collection during both measurements, the schools were visited by the research team. After a short introduction by the researcher, children and teacher completed a questionnaire in their classroom; this took approximately 30 min. During the second follow-up measurement, the same procedure was followed for nine schools, whereas in three other schools the questionnaires were distributed by the teachers themselves because the original classes had been split or the schools had no time for a visit from the research team. Children completed a questionnaire on behavioural determinants (outcome measures) at all three time points and an additional appreciation questionnaire during the first follow-up measurement. For the latter questionnaire, the teacher recapitulated the lessons that the children had received to help them recall which activities were part of which lesson. Teachers completed a questionnaire on their background characteristics at baseline and a questionnaire on programme delivery and appreciation at the first follow-up measurement. Children took part in the study whose parents did not object to their participation.

### Measures

#### Process indicators

The process evaluation questionnaires for both the teachers and the children included closed and open questions, and were based on questionnaires used for the evaluation of the Dutch nutrition education programme *Krachtvoer* [23]. A translation of the questionnaires could be find in the Additional file 1.

##### Dose

Teachers could indicate on a checklist for each lesson which standard activities they had implemented. The proportion of implemented standard activities was calculated for each class by dividing the total number of implemented activities by the total number of activities on the curriculum.

##### Appreciation

For each implemented lesson, the teacher’s questionnaire measured the extent to which the teachers appreciated the lesson (10-point scale), how much they liked the lesson and how feasible the lesson was to implement (both on a 5-point scale, ranging from 1 = not nice/not feasible to 5 = very nice/feasible, respectively). In addition, more detailed information on programme delivery, such as perceived constraints and opinion on the programme materials, was assessed with open questions. For each implemented lesson, the children’s questionnaire measured the extent to which they liked the lesson (10-point scale). Additionally, the questionnaire assessed appreciation of specific activities (e.g. taste-testing and home assignments, 10-point scale) and how they appreciated Taste Lessons in general (5-point scale, ranging from 1 = not nice to 5 = very nice).

##### Interpersonal communication

One question on the children’s appreciation questionnaire assessed how often children talked about Taste Lessons with others after the lessons (5-point scale, ranging from 1 = never to 5 = always).

#### Behavioural determinants

The outcome measure of the Taste Lessons effect evaluation was children’s change in behavioural determinants towards tasting unfamiliar products and eating healthy and a variety of foods. The behavioural determinants selected were knowledge, awareness, skills, attitude, emotion, subjective norm (of classmates, parents and teacher) and intention. Children’s knowledge (six questions, scale true or false) and skills (four questions, able to perform the skill, scale ‘no’, ‘a little’ or ‘yes’) were assessed by questions on what they were taught during Taste Lessons. Awareness was measured by questions on how often children performed the target behaviours (5-point scale, ranging from 1 = never to 5 = always). Questions and scales for attitude and emotion (‘how much do you think the target behaviours are clever/interesting and nice/cool/tasty?’), subjective norm (’how much do you think your classmates/parents/ teacher want you to perform the target behaviours?’) and intention (‘how much are you planning to perform the target behaviours?’) were used as described by Ajzen and Fishbein [24] (5-point scale, ranging from 1 = no, not at all to 5 = yes, totally). A questionnaire was developed to be filled out by the children themselves and pretested. Reliability analyses of the baseline data showed Cronbach's alpha >0.6 for all constructs, and mean scores were used in further analyses. For knowledge, the criteria of the facility index and item discrimination were used to exclude questions, and the score for correct answers was used for further analyses [25]. Change scores were calculated as the difference between the children’s mean score at the baseline measurement and at the follow-up measurements [26].

#### Socio-demographic characteristics

The children’s questionnaire at baseline included questions on age (in years), sex and ethnicity of the children and their parents (country of birth). Children were classified as non-native if they or one of their parents were born outside the Netherlands. In the teacher’s questionnaire at baseline, sex and years of teaching experience were assessed. Information on the schools’ characteristics was obtained from the online database of Dutch elementary schools [27], including location (city, small city or town), school type (religious or public) and school size (small [<150 pupils], medium [150–400 pupils] or large [>400 pupils]).

### Statistical analysis

Descriptive analyses were performed, using SPSS Statistics (version 19.0) to describe the socio-demographic characteristics and process indicators. Subsequently, the association between the process indicators and change in behavioural determinants was assessed for both follow-up measurements compared to baseline, for grades 5–8 together. Multivariate linear regression analyses were performed by use of the programme HLM (version 7) to adjust for a clustering effect of children within the same class and school, including three levels: (1) pupil, (2) class and (3) school. Changes in the behavioural determinant scores were used as dependent variables. The process indicators dose (proportion of standard activities received, score 0–1), teacher and children appreciation (mean score on liking of Taste Lessons, both scale 1–5) and the extent to which children talked about the lessons (mean score on interpersonal communication, scale 1–5) were used as explanatory variables in separate analyses. Analyses were adjusted for children’s sex and baseline age. Results were interpreted as significant when *p* < 0.05.

## Results

### Characteristics of the study population

The study sample consisted of 392 children (224 children in grades 5–6, 168 children in grades 7–8), with a mean age of 9.6 years at baseline (Table 1). Of the 20 teachers that implemented Taste Lessons, most were female (90 %) and had on average 16.7 years of teaching experience.

**Table 1:** Descriptive statistics of the study population by children, teacher and school characteristics

	Total		Grades 5–6		Grades 7–8	
	N	Mean (SD) or %	N	Mean (SD) or %	N	Mean (SD) or %
Children characteristics (*n* = 392)						
Age (years)	392	9.6 (1.3)	224	8.7 (0.7)	168	10.8 (0.7)
Sex – girls	179	45.7	107	47.8	72	42.9
Ethnicity – native^a^	247	63.0	145	64.7	102	60.7
Teacher characteristics (*n* = 20)^b^						
Sex – female	18	90.0	11	84.6	6	75.0
Teaching experience (years)^c^	18	17.1 (13.4)	11	19.0 (14.6)	8	14.4 (11.9)
School characteristics (*n* = 392)						
*Location*						
- Village (<10,000 inhabitants)	129	32.9	97	43.3	32	19.0
- Small city (10,000–100,000 inhabitants)	211	53.8	111	49.6	100	59.5
- City (>100,000 inhabitants)	52	13.3	16	7.1	36	21.4
*School type*						
- Public	106	27.0	47	21.0	59	35.1
- Religious	286	73.0	177	79.0	109	64.9
*School size*						
- Small (<150 students)	145	37.0	71	31.7	74	44.0
- Medium (150–400 students)	247	63.0	153	68.3	94	56.0

^a^Grades 5–8: 28 missing (7 %), grades 5–6: 5 missing (2 %), grades 7–8: 23 missing (14 %)

^b^One male teacher had a grade 6–7 class

^c^Two teachers’ data are missing

### Programme delivery

Teachers implemented on average 4.6 lessons and 28 % of the activities (Table 2). Grades 5–6 teachers implemented on average more lessons and activities than grades 7–8 teachers. Furthermore, lessons at the beginning of the curriculum were more often implemented than lessons at the end of the curriculum.

**Table 2:** Teacher’s and children’s score on the process indicators dose, appreciation and interpersonal communication

	Total		Grades 5–6		Grades 7–8	
	N	Mean (SD)	N	Mean (SD)	N	Mean (SD)
Teacher questionnaire (*n* = 18)						
*Dose*						
- Number of lessons^a^	18	4.6 (3.2)	10	5.5 (4.1)	8	3.5 (1.2)
- Number of activities^b^	18	18.9 (16.0)	10	25.3 (19.0)	8	10.9 (5.3)
- Proportion of activities (0–1)^c^	18	0.28 (0.21)	10	0.34 (0.25)	8	0.21 (0.1)
*Appreciation*						
- Score (1–10)	18	7.9 (0.8)	10	7.7 (0.7)	8	8.3 (0.9)
- Liking (1–5)	18	4.4 (0.4)	10	4.4 (0.4)	8	4.4 (0.5)
- Feasible (1–5)	18	4.1 (0.7)	10	3.9 (0.7)	8	4.3 (0.6)
Child questionnaire (n = 339)						
*Appreciation*						
- Score (1–10)	339	7.9 (2.0)	199	8.5 (1.8)	140	7.2 (2.0)
- Liking (1–5)	331	4.0 (1.0)	180	4.3 (0.8)	151	3.6 (1.1)
*Appreciation of activities (score 1–10)*						
- Taste-testing	334	8.5 (2.2)	191	8.9 (1.8)	143	7.9 (2.4)
- Conducting experiments	307	8.7 (1.8)	181	9.0 (1.7)	126	8.3 (1.9)
- Looking for information	199	6.3 (2.5)	97	6.5 (2.8)	102	6.2 (2.2)
- Talking about nutrition	309	6.6 (2.4)	174	7.1 (2.5)	135	6.0 (2.1)
- Learning about taste and food	306	7.1 (2.4)	174	7.9 (2.2)	134	6.0 (2.4)
*Interpersonal communication (1–5)* ^d^	331	2.8 (1.1)	180	2.8 (1.1)	151	2.7 (1.1)

^a^Lessons for grades 5–6 range from 1–12, for grades 7–8 from 1–10

^b^Activities for grades 5–6 range from 1–75, for grades 7–8 from 1–52

^c^The total number of implemented activities divided by the total number of activities on the curriculum

^d^How often the children talked about Taste Lessons with others after the lessons

Teachers were positive about Taste Lessons, with a mean score of 7.9 (Table 2) and perceived the lessons to be nice and feasible to implement. Teachers perceived the Taste Lessons materials and curriculum as attractive, informative, and a good way to keep children engaged and to teach them about food and nutrition. Children were positive about the Taste Lessons as well (mean score 7.8). Children from grades 5–6 had a higher appreciation of Taste Lessons than children from grades 7–8. In general, children were most positive about practical activities such as taste-testing and conducting experiments. Theoretical activities were rated lower but still positively, with mean scores between 6.3 and 7.1.

Children scored moderately on interpersonal communication, with similar scores for grades 5–6 and grades 7–8. Most of the children talked (almost) never (40 %) or sometimes (37 %) about the lessons afterwards, whereas 24 % of the children talked (almost) always with others about Taste Lessons after they had taken place.

### Association between the process indicators and the programme outcomes

At the first follow-up measurement, positive associations were found between dose and all behavioural determinant change scores, but a significant dose–response relation was only shown for change in subjective norm of the teacher (*p* < 0.03, Table 3). At the second follow-up measurement, the general trend in associations was still positive, but no significant associations were found.

**Table 3:** Associations between the process indicators and changes in behavioural determinants^a^

	*Dose*		*Appreciation (teacher)*		*Appreciation (children)*		*Interpersonal communication*	
	1st follow-up – baseline (*N* = 338)	2nd follow-up – baseline (*N* = 249)	1st follow-up – baseline (*N* = 338)	2nd follow-up – baseline (*N* = 249)	1st follow-up – baseline (*N* = 296)	2nd follow-up – baseline (*N* = 206)	1st follow-up – baseline (*N* = 296)	2nd follow-up – baseline (*N* = 205)
	β (SE)	β (SE)	β (SE)	β (SE)	β (SE)	β (SE)	β (SE)	β (SE)
Knowledge	0.02 (0.09)	−0.01 (0.10)	−0.06 (0.04)	−0.09 (0.04)	−0.01 (0.02)	0.01 (0.02)	0.02 (0.01)*	0.00 (0.01)
Awareness	0.56 (0.28)	0.37 (0.41)	−0.24 (0.12)	−0.40 (0.16)	0.12 (0.05)*	0.12 (0.07)	0.09 (0.04)*	0.09 (0.05)
Skills	0.00 (0.13)	0.05 (0.17)	0.05 (0.05)	−0.03 (0.09)	−0.02 (0.03)	0.00 (0.04)	0.00 (0.02)	0.00 (0.03)
Attitude	−0.05 (0.26)	−0.48 (0.32)	−0.24 (0.09)*	−0.16 (0.17)	0.10 (0.05)	0.20 (0.07)**	0.18 (0.04)***	0.16 (0.06)**
Emotion	0.10 (0.25)	0.41 (0.33)	−0.15 (0.10)	−0.14 (0.16)	0.13 (0.05)**	0.13 (0.07)	0.20 (0.04)***	0.09 (0.06)
Subjective norm – classmates	0.27 (0.37)	0.01 (0.50)	−0.12 (0.16)	−0.46 (0.22)	0.08 (0.07)	0.14 (0.10)	0.10 (0.06)	0.08 (0.08)
Subjective norm – parents	0.41 (0.18)	0.13 (0.27)	−0.07 (0.08)	−0.05 (0.14)	0.05 (0.04)	0.12 (0.06)*	0.04 (0.03)	−0.02 (0.05)
Subjective norm – teacher	1.19 (0.39)*	0.95 (0.38)	−0.47 (0.16)*	−0.22 (0.21)	0.20 (0.07)**	0.10 (0.09)	0.07 (0.06)	−0.06 (0.08)
Intention	0.05 (0.23)	−0.31 (0.38)	−0.06 (0.10)	−0.31 (0.18)	0.06 (0.05)	0.11 (0.08)	0.17 (0.04)***	0.18 (0.06)**

^a^For grades 5–8 together, with children who filled in at least 75 % of the questions in the constructs, and adjusted for children’s sex and baseline age

* *p* < 0.05, ** *p* <0.01, *** *p* <0.001

Teacher appreciation showed inverse associations with change in almost all determinants at both measurements, of which change in attitude and subjective norm of the teacher at the first follow-up measurement were significant (both *p* < 0.05). An opposite trend was observed for children. The better the children appreciated Taste Lessons, the more positive change they showed in the determinants. Children’s appreciation was significantly positively associated with change in awareness (*p* < 0.05), emotion (*p* < 0.01) and subjective norm of the teacher (*p* < 0.01) at the first follow-up measurement. Six months after the intervention, these significant positive associations were still significant for change in attitude (*p* < 0.01) and subjective norm of the parents (*p* < 0.05).

Interpersonal communication was positively related to almost all determinants at both follow-up measurements. Significant associations were found for change in knowledge and awareness (both *p* < 0.05), and attitude, emotion and intention (all *p* < 0.001) one month after Taste Lessons. During the second follow-up measurement, talking about Taste Lessons remained significantly positively associated with change in attitude and intention (both *p* < 0.01).

## Discussion

The aim of this study was to provide insight into the programme delivery of Taste Lessons, and to investigate the extent to which process indicators could indicate programme effect on behavioural determinants. Taste Lessons was positively appreciated by both teachers and children. Mainly due to time and money constraints however, teachers implemented only some of the lessons and activities. Process indicators reflecting different aspects of the programme delivery were associated with the measured outcomes differently. Children’s appreciation and interpersonal communication both showed significant positive associations with change in awareness, attitude and emotion, whereas children’s appreciation and dose were both significantly associated with subjective norm of the teacher. In addition, interpersonal communication was significantly associated with change in children’s knowledge and intention towards tasting unfamiliar food and eating healthy and a variety of foods. Remarkably, teacher appreciation was negatively associated with changes in determinants.

Before the results of this study are discussed, there are a number of limitations that should be taken into account. The most important limitation is that all the data in this study were collected using self-report, and this may have led to socially desirable answers and measurement errors. However, to reduce measurement errors, questions and answers were formulated to be child-friendly and the questionnaire was pretested. Furthermore, children completed the questionnaires under supervision of the research team, who also instructed the children on how to fill in the questionnaires and were available for questions.

A second limitation in this study could be that process indicators and effect were studied in a real-life setting, without strict guidelines for teachers about which lessons or activities to implement. Teachers were instructed on how to implement Taste Lessons during the introductory workshop. They were, however, free to implement Taste Lessons in either a project week or lessons spread over a longer period of time and were not obliged to implement all the programme lessons and activities.

In general, this resulted in high appreciation and feasibility, but incomplete implementation of the programme, with a mean delivered dose of around one-third of the available activities and 35 % to 45 % of the lessons implemented in the classroom. In other studies, a higher delivered dose of activities is reported, ranging from 47 % to 91 % of activities implemented [9, 28–32] or 45 % to 95 % of the curriculum lessons implemented [10–12, 31]. However, those programmes were implemented in other countries, which might have different nutrition education policies than The Netherlands. Dutch schoolteachers have limited time for nutrition education, as nutrition education is not mandatory in Dutch primary schools. The programmes where more than 80 % of the lessons or activities were implemented were all conducted in the USA [9, 10, 28, 31]. In the British programme ‘Project Tomato’, 45 % of the lessons were implemented, which is only a little more than Taste Lessons in the current study. Besides, the programmes were in some studies delivered by research staff [28], and teachers in all other programmes may have been stimulated to implement the whole curriculum.

The received dose of Taste Lessons was a predictor of change in subjective norm of the teacher only. It is plausible that the more teachers taught children about nutrition, the more children perceived that the teacher wanted them to taste unfamiliar products and eat healthy and a variety of foods. This study found no significant relation between self-reported dose and other determinants, such as knowledge. A study on the programme Gimme 5 found a positive association between interview-assessed dose and health knowledge, but no association between self-reported dose and health knowledge [9]. Gray et al.’s study [12] found significantly higher increases in behavioural determinants related to both decreasing unhealthy behaviours and increasing healthy behaviours, such as self-efficacy and intention, in classes with a higher delivered dose than in classes with a lower delivered dose. Other studies found positive [7] or no associations [8, 10] between delivered dose and the behavioural outcome fruit and vegetable intake. Durlak and DuPre [15] suggest that it is not realistic to expect complete implementation, but positive results have often been obtained with around 60 % implementation. It could also be that using only a number of activities as a measure of dose and not a certain type of activity (such as taste-testing or other practical activities) could have underestimated the dose–response effect. More research has to be done to explore whether there is a threshold of exposure to (certain elements of) the programme required to achieve desired effects.

Teacher appreciation of Taste Lessons was positive in this study, but the more positive teachers were about the curriculum, the less positive changes were found in children’s behavioural determinants. Teachers who were contacted to discuss these remarkable findings could not provide an explanation for this effect. To our knowledge, only Christian et al.’s evaluation study [11] on Project Tomato assessed the relation between teacher appreciation and programme effects, and no relation was found. However, teacher appreciation (satisfaction with both the curriculum materials and teaching the curriculum) significantly correlated with student satisfaction in Gray et al.’s study [12]. In their evaluation model, Gray et al. hypothesised that teacher appreciation links to delivered dose and children’s engagement and appreciation, but not directly to programme outcomes [12]. Teacher appreciation might therefore not be the right (direct) indicator for effectiveness of the programme. This hypothesis might be supported by Dusenbury et al.’s finding [16] that teacher self-reports about adaptations to the programme negatively correlated to observations.

Children’s appreciation in our study was positively associated with change in behavioural determinants. Especially strong associations were found with attitude and emotion, which are determinants that are closely linked to perceptions of liking. Other studies that have looked at the relation between children’s appreciation and programme effect found either significant positive associations using categorical appreciation scores [7, 8, 12] or no significant associations using a continuous appreciation score [11]. Positive children’s appreciation seems therefore to be more indicative of programme effects than teacher appreciation.

In the current study, significant associations were found between interpersonal communication about Taste Lessons and the change in most of the selected behavioural determinants, especially after one month. To our knowledge, talking about a programme has been extensively studied only in mass media campaigns [18–20]. In particular, emotionally engaging messages or activities are promising means to increase the likelihood of conversation, but the topic and the person that is talked with are important factors for obtaining effect [19]. Intervention effects of Taste Lessons via interpersonal communication may be enhanced if attractive activities, such as experiments, are used, and if teachers are able to get the children fascinated by the topic. This overlaps with enthusiasm of the teacher and engagement of the children as potential factors for influencing implementation [12, 15, 16]. The results in the current study were obtained from a single question on conversational occurrence. Conversation content and which persons were talked with were not assessed. Further research is therefore necessary to unravel the relation between interpersonal communication and programme outcomes.

## Conclusions

From the results it can be concluded that delivered dose, children’s appreciation and interpersonal communication are indicators of a positive programme effect on behavioural determinants towards tasting unfamiliar foods and eating healthy and a variety of foods. Therefore, process evaluation provides insight into factors that contribute to the effect of interventions in real-life settings.

## Additional file

Additional file 1: A translation of the questionnaires. (DOCX 74 kb)
