# Supplementary material for: Unravelling the effect of the Dutch school-based nutrition programme Taste Lessons: the role of dose, appreciation and interpersonal communication
Source: BMC Public Health. 2016 Aug 5;16:737. doi: 10.1186/s12889-016-3430-1 (PMC4975919; doi:10.1186/s12889-016-3430-1)
Supplement: Additional file 1: — A translation of the questionnaires. (DOCX 74 kb) [file 12889_2016_3430_MOESM1_ESM.docx]

## Child effect evaluation questionnaire

**General information**

1. What is your name?

My name is: …………………………………………………………

2. Are you a girl or a boy?

- Boy
- Girl

3. How old are you?

I am …… years old

4. In what country were you born?

- The Netherlands
- Other: .................................

5. In what country was your mother born?

- The Netherlands
- Other: .................................

6. In what country was your father born?

- The Netherlands
- Other: .................................

**Knowledge**

|  | **True** | **False** | **I don’t know** |
| --- | --- | --- | --- |
| 1. You can taste with your tongue whether there is salt in a product | 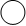 | 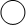 | 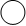 |
| 1. It doesn’t matter whether you have a stuffy nose or not, because you can always taste the same amount | 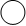 | 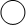 | 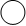 |
| 1. When you taste unfamiliar products more often, more products will be tasty | 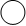 | 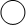 | 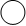 |
| 1. It is unhealthy to eat different products every day | 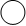 | 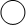 | 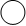 |
| 1. You need to eat products from the Wheel of Five to stay healthy | 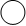 | 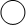 | 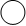 |
| 1. A bit of salt enhances the other flavours in your food | 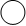 | 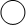 | 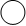 |

**Intention**

|  | **Yes,**  **certainly** | **Yes, I think so** | **In between** | **No, I don’t think so** | **No, certainly not** |
| --- | --- | --- | --- | --- | --- |
| 1. I have plans more often to taste products that I have never eaten before | 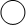 | 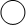 | 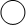 | 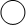 | 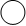 |
| 1. I have plans more often to taste again products that I don’t like at this moment | 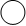 | 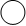 | 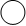 | 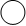 | 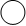 |
| 1. I have plans to eat and drink healthy products more often | 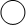 | 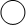 | 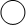 | 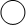 | 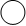 |
| 1. I have plans to eat and drink a variety of foods more often | 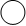 | 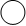 | 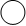 | 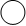 | 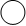 |

**Awareness**

|  | **Always** | **Regularly** | **Sometimes** | **Rarely** | **Never** |
| --- | --- | --- | --- | --- | --- |
| 1. When I’m going to eat something, I pay attention to whether it looks tasty | 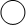 | 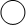 | 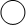 | 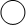 | 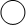 |
| 1. When I’m going to eat something, I pay attention to whether it smells nice | 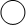 | 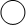 | 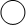 | 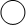 | 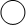 |
| 1. When I’m going to eat something, I pay attention to whether it’s healthy | 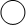 | 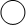 | 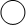 | 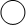 | 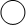 |
| 1. When I eat something, I try to taste it well |  |  |  |  |  |
| 1. When I eat something, I pay attention to whether it tastes sweet, sour, bitter or salty | 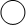 | 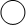 | 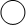 | 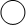 | 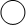 |
| 1. I pay attention to whether I eat different products every day | 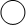 | 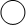 | 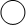 | 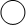 | 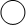 |
| 1. I pay attention to whether I eat a lot of basic products from the Wheel of Five | 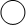 | 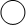 | 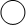 | 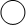 | 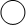 |

**Attitude & emotion**

1. How do you think about tasting products that you’ve never eaten before?

|  | **Yes,**  **certainly** | **Yes, I think so** | **In between** | **No, I don’t think so** | **No, certainly not** |
| --- | --- | --- | --- | --- | --- |
| I think that’s interesting | 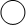 | 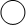 | 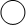 | 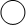 | 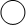 |
| I think that’s clever | 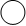 | 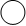 | 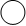 | 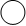 | 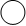 |
| I think that’s nice | 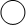 | 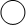 | 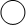 | 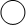 | 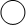 |
| I think that’s tasty | 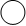 | 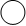 | 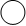 | 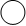 | 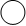 |
| I think that’s cool | 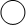 | 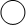 | 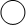 | 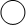 | 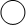 |

2. How do you think about tasting products again later that you don’t like at this moment?

|  | **Yes,**  **certainly** | **Yes, I think so** | **In between** | **No, I don’t think so** | **No, certainly not** |
| --- | --- | --- | --- | --- | --- |
| I think that’s interesting | 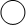 | 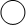 | 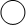 | 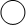 | 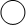 |
| I think that’s clever | 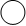 | 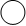 |  |  |  |
| I think that’s nice |  |  |  |  |  |
| I think that’s tasty |  |  |  |  |  |
| I think that’s cool |  |  |  |  |  |

3. How do you think about consuming healthy foods and drinks?

|  | **Yes,**  **certainly** | **Yes, I think so** | **In between** | **No, I don’t think so** | **No, certainly not** |
| --- | --- | --- | --- | --- | --- |
| I think that’s interesting |  |  |  |  |  |
| I think that’s clever |  |  |  |  |  |
| I think that’s nice |  |  |  |  |  |
| I think that’s tasty |  |  |  |  |  |
| I think that’s cool |  |  |  |  |  |

4. How do you think about consuming different foods and drinks every day?

|  | **Yes,**  **certainly** | **Yes, I think so** | **In between** | **No, I don’t think so** | **No, certainly not** |
| --- | --- | --- | --- | --- | --- |
| I think that’s interesting |  |  |  |  |  |
| I think that’s clever |  |  |  |  |  |
| I think that’s nice |  |  |  |  |  |
| I think that’s tasty |  |  |  |  |  |
| I think that’s cool |  |  |  |  |  |

**Subjective norm**

|  | **Yes,**  **certainly** | **Yes, I think so** | **In between** | **No, I don’t think so** | **No, certainly not** |
| --- | --- | --- | --- | --- | --- |
| My classmates want me to taste products that I’ve never eaten before |  |  |  |  |  |
| My classmates want me to taste products again later that I don’t like at this moment |  |  |  |  |  |
| My classmates want me to consume healthy foods and drinks |  |  |  |  |  |
| My classmates want me to consume different foods and drinks every day |  |  |  |  |  |

|  | **Yes,**  **certainly** | **Yes, I think so** | **In between** | **No, I don’t think so** | **No, certainly not** |
| --- | --- | --- | --- | --- | --- |
| My parents want me to taste products that I’ve never eaten before |  |  |  |  |  |
| My parents want me to taste products again later that I don’t like at this moment |  |  |  |  |  |
| My parents want me to consume healthy foods and drinks |  |  |  |  |  |
| My parents want me to consume different foods and drinks every day |  |  |  |  |  |

|  | **Yes,**  **certainly** | **Yes, I think so** | **In between** | **No, I don’t think so** | **No, certainly not** |
| --- | --- | --- | --- | --- | --- |
| My teacher wants me to taste products that I’ve never eaten before |  |  |  |  |  |
| My teacher wants me to taste products again later that I don’t like at this moment |  |  |  |  |  |
| My teacher wants me to consume healthy foods and drinks |  |  |  |  |  |
| My teacher wants me to consume different foods and drinks every day |  |  |  |  |  |

**Skills**

|  | **Yes** | **A little** | **No** |
| --- | --- | --- | --- |
| I’m able to cook using a recipe |  |  |  |
| I’m able to cook together with others |  |  |  |
| I'm able to prepare a healthy lunch |  |  |  |
| I'm able to recognise tastes |  |  |  |

## Child process evaluation questionnaire

How much did you like Taste Lessons?

Mark (1–10): …………………

How much did you like the activities of Taste Lessons?

|  | Mark |
| --- | --- |
| Taste-testing |  |
| Conducting experiments |  |
| Looking for information |  |
| Talking about nutrition |  |
| Learning about taste and food |  |

How nice or annoying do you think Taste Lessons was?

□ Very nice

□ Nice

□ Not nice, not annoying

□ Annoying

□ Very annoying

How often did you talk about Taste Lessons with others after the lessons?

□ Always

□ Almost always

□ Sometimes

□ Almost never

□ Never

## Teacher process evaluation questionnaire

*The same format for each lesson. An example of lessons 1 for grades 5–6:*

Have you implemented lesson 1? □Yes □ Partly □ No

If not, why not?

…………………………………………………………………………………………………

…………………………………………………………………………………………………

Which activities of this lesson have you implemented?

□ 1. Let the children fill out and evaluated the first assignment of the taste passport

□ 2. Talked about ‘why do we eat?’

□ 3. Let the children fill out and evaluate the second assignment of the taste passport

□ 4. Let the children taste products which taste sweet, sour, salty and bitter

*Which products?*

…………………………………………………………………………........

□ 5. Asked questions about the senses you use when tasting

□ 6. Let the children taste products and fill out copy sheet 1

*Which products?*

…………………………………………………………………………........

□ 7. Let the children fill out and evaluate the third assignment of the taste passport

□ A. Let the children taste products on a dry tongue

*Which products?*

…………………………………………………………………………........

□ B. Let the children feel products and let them describe what they feel

*Which products?*

…………………………………………………………………………........

□ C. Let the children hear products and discuss the effects on their appreciation of the
product

*Which products?*

…………………………………………………………………………........

□ Other activities:

………………………………………………………………………………..

………………………………………………………………………………..

What is your opinion about this lesson?

Very feasible to implement 1 2 3 4 5 Not feasible to implement

Very nice 1 2 3 4 5 Not nice

What mark would you give this lesson?

Mark (1–10): ………………………………
